# Contamination of beef products with staphylococcal classical enterotoxins in Egypt and Saudi Arabia

**DOI:** 10.3205/dgkh000268

**Published:** 2016-04-01

**Authors:** Reyad R. Shawish, Naser A. Al-Humam

**Affiliations:** 1 Department of Food Hygiene & Control, Faculty of Veterinary Medicine, University of Sadat City, Egypt; 2 Department of Microbiology and Parasitology, Faculty of Veterinary Medicine, King Faisal University, Al-Ahsa, Kingdom of Saudi Arabia

**Keywords:** Staphylococcus aureus, meat products, staphylococcal enterotoxins, PCR, Egypt, Kingdom of Saudi Arabia

## Abstract

Food-borne pathogens are of high concern for public health and food safety. *Staphylococcus aureus* food poisoning is one of the most economically devastating types of food poisoning globally. The purpose of this study was to detect staphylococcal classical enterotoxins (SEs) in processed beef from Kingdom of Saudi Arabia (KSA) and Egypt.

In the present investigation a total of 250 random processed meat samples (50 each of minced meat, beef burger, beef sausage, beef kofta and beef luncheon) were collected from different super markets in the study area. Using conventional cultural methods, samples were cultured for isolation and identification of *S. aureus*. Multiplex PCR was used to detect SEs of the classical type SEA, SEB, SEC and SED from isolates.

The percentage presence of *S. aureus* in minced meat, beef burger, beef sausage, beef kofta and beef luncheon was 38%, 22%, 30%, 32% and 12%, respectively. Multiplex PCR indicated that all examined samples contain different types of classical staphylococcal enterotoxins and only minced meat samples contained all four types of toxins.

Multiplex PCR is efficient in detection of SEs from food and may be used in tracing of toxins to promote food hygiene. Implications of contamination of processed meat to food hygiene in the study area are highlighted.

## Zusammenfassung

Lebensmittelpathogene sind von großer Bedeutung für die öffentliche Gesundheit und die Lebensmittelsicherheit. *Staphylococcus aureus* ist einer der ökonomisch bedeutsamsten Lebensmittelvergifter weltweit. Zielsetzung der vorliegenden Arbeit war der Nachweis von klassischen Staphylokokkenenterotoxinen (SE) in Rindfleischerzeugnissen im Königreich Saudi-Arabien (KSA) und in Ägypten.

250 Proben, davon je 50 aus Hackfleisch, Rindfleisch-Burger, Wurst, Rindfleischbällchen (Kofta) und Rindfleischfertigessen, wurden randomisiert in verschiedenen Supermärkten in der Studienregion gezogen. Mittels konventioneller kultureller Diagnostik wurde *S. aureus* isoliert und identifiziert. Zum Nachweis der klassischen SE-Typen SEA, SEB, SEC und SED wurde die Multiplex PCR eingesetzt.

*S. aureus* wurde in 38% der Hackfleischproben, 22% der Rindfleisch-Burger, 30% der Würste, 32% der Rindfleischbällchen (Kofta) und 12% des Rindfleischfertigessens nachgewiesen. Alle untersuchten Proben enthielten verschiedene SE-Typen; nur Hackfleisch enthielt alle vier Typen.

Die Multiplex PCR ist ein geeignetes Verfahren zum Nachweis von SE in Lebensmitteln und kann zur Verbesserung der Lebensmittelhygiene eingesetzt werden. Anhand der Befunde werden die Auswirkungen der Kontamination von verarbeitetem Fleisch auf die Lebensmittelhygiene hervorgehoben.

## Introduction

Food poisoning caused by *Staphylococcus aur*eus is an intoxication mediate by enterotoxins contaminating food. *S. aureus* is considered the world’s third most important cause of food-borne diseases. The ability of *S. aureus* to grow and produce toxins under different environmental and nutritional conditions is evident from the variety of foods implicated in staphylococcal food poisoning. Frequently incriminated foods in staphylococcal food poisoning include meat, meat products, milk and dairy products and poultry meat and eggs. The demand for convenient, ready-to-eat fast food has increased recently. In response to this, the food industry has developed new methods for food processing such as chilled food, semi-prepared, minimally processed meals [1].

 In Egypt and the Kingdom of Saudi Arabia (KSA) meat products such as sausage, beef burger and luncheon are gaining popularity because they represent quick easily prepared meat meals and solve the problem of fresh and expensive meat shortage, which is not within the income reach of many families with limited income. 

*S. aureus* is involved in a variety of invasive diseases in humans and animals; in human it is a significant pathogen in causing nosocomial infection. Conversely, humans act as a reservoir of *S. aureus*, since this bacteria may be carried in nares and on hands, and does not always cause disease [2]. Humans carrying enterotoxin-producing *S. aureus* on their hands or in their nostrils are regarded as the main source of food contamination, via mechanical contact or through aerosol droplets, if involved in food processing. 

Staphylococcal enterotoxins (SEs) are classified as members of the pyrogenic toxin super-antigen family because of their related biological activities and structural relatedness. Emetic activities have been proposed as criteria to designate super-antigens (SAgs) as SEs only after experimental induction of vomiting by SAgs orally administered in a primate model [3]. Activity of SEs in high nanogram to low microgram concentrations was observed [4]. SEs function both as potent gastrointestinal toxins, and SAgs that stimulate nonspecific T-cell proliferation. These two functions are localized on separate molecular regions within the proteins, and a loss of super-antigenic function through mutagenesis does not necessarily result in abrogation of enterotoxic activity. They are resistant to heat treatment, low pH and proteolytic enzymes hence retaining their activity in the digestive tract after ingestion [5]. The existence of SEs has been reported to range from SEA to SEV: the classical types SEA to SEE and the new types SEG to SEV.

A study on the impact of food-related illness in the USA was done due to a number of serious incidents in recent years. Consequently, the US Food and Drug Administration (FDA) has increased efforts to survey and improve the quality of contaminated products [6]. In Europe, the EFSA reported a total of 5,550 outbreaks of foodborne illness in 2009, affecting almost 49,000 people and causing 46 deaths. Among these, 293 outbreaks were caused by *Staphylococcus* spp. and bacterial toxins (produced by *Bacillus*, *Clostridium* and *Staphylococcus)* were the fourth most common causative agent in foodborne diseases [7]. 

The symptoms of *S. aureus* intoxication are severe vomitus, stomach cramps and diarrhea, sometimes followed by collapse. The incubation period of *S. aureus* food poisoning was reported as low as 2–6 hrs, and duration of illness 6–24 hrs [8].

The aim of the present study was to determine the occurrence of SEs and virulence gene profile of *S. aureus* isolated from beef products samples collected at the retail level in Egypt and KSA.

## Material and methods

### Sample collection

Processed meat samples were collected from different types of supermarkets. These establishments ranged from small grocery to large supermarket from June 1 to June 30 of the same year. A total of 25 samples each from minced meat, beef burger, beef sausage, beef kofta and beef luncheon were collected from different super markets at Menofia, and Cairo governorates, Egypt. The same number and type of food products were collected from Al-Ahsa Province, KSA during the same period. All samples were collected during daytime when outdoor temperature ranged from 29°C to 33°C in both study areas. Only one sample each was collected from each location. The samples were transferred immediately after sampling in a cooling ice box to the laboratory. Each sample was mixed to ensure homogeneity. Sample homogenate was prepared by homogenizing 10 g of each sample separately in 90 ml of tryptic soy broth for 2 min using stomacher in sterile polyethylene bags then 15 ml of each sample were transferred to sterile screw capped tube. The samples were incubated at 37°C for 24 hours and then cultivated.

### Isolation and identification of S. aureus

The prepared incubated samples were streaked on the surface of Baird-Parker agar base (Oxoid, CM 0275, UK). The inoculated plates were incubated for 24–48 hours at 37°C after which they were examined for colony characters, cellular morphology and the purity of the culture. The biochemical and cultural characters of the suspected colonies were studied according to [9].

### Multiplex polymerase chain reaction (PCR) for detection of S. aureus superantigen

Pure strains of *S. aureus* were cultured on brain heart infusion broth and incubated overnight then used for DNA extraction [10].

### Genomic DNA extraction

Chromosomal DNA was isolated from STSE isolates using GeneJET Genomic DNA Purification Kit (Fermentas).

### DNA amplification for multiplex PCR reaction

Multiplex PCR reaction was performed and each reaction mixture contained 1 µl of prepared template DNA, 0.5 µmol of each primer (Table 1 (Tab. 1)), 25 µL of 2x multiplex master mix (QIAGEN) and the final volume was adjusted to 50 µL with distilled water. PCR was performed in rotor gene thermocycler with the following steps: 94°C for 30 s, 55°C for 30 s, 72°C for 2 min with final extension at 72°C for 10 min. The amplified products were resolved by electrophoresis in 2% agarose gel at 100 V for 60 min.

### Separation of PCR amplicons by gel electrophoresis

After the amplification was completed the amplified products were analyzed on agarose gel (consisting of 2% agarose and 5 µL of ethidium bromide in 1x Tris-Acetate EDTA (TAE) buffer. The samples were electrophoresed at 100 volts for one hour and examined under ultraviolet transiluminator and photographed.

## Results

Investigation on the presence of *S. aureus* in meat products showed that 19 out of 50 minced meat samples, 11 of beef burger, 15 of beef sausage, 16 of beef kofta and 6 of beef luncheon contained the species. As for the strains that produced SEs were 8 out of 19 isolates in minced meat, 4 from 11 isolates in beef burger, 4 from 15 isolates in beef sausage, 3 from 16 isolates in beef kofta and 1 from 6 isolates in beef luncheon (Table 2 (Tab. 2)). No difference was observed in samples from KSA and Egypt. 

**Figure 1:**
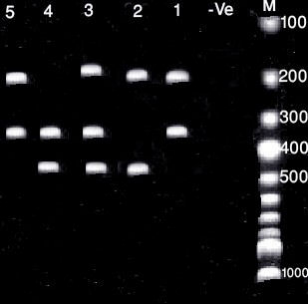
Agarose gel electrophoresis show multiplex PCR products of *S. aureus* strains (staphylococcal enterotoxin A gene 344 bp, staphylococcal enterotoxin B gene 196 bp, staphylococcal enterotoxin C gene 399 bp and staphylococcal enterotoxin D gene 451 bp). Lane 1 contains enterotoxin A and B genes. Lane 2 contains enterotoxin B and D genes. Lane 3 contains enterotoxin A, B and D genes. Lane 4 contains enterotoxin A and D genes. Lane 5 contains enterotoxin A and B genes

Multiplex PCR reaction was carried out and resolved by agarose gel electrophoresis. It showed products of staphylococcal enterotoxin A gene 344 bp, staphylococcal enterotoxin B gene 196 bp, staphylococcal enterotoxin C gene 399 bp and staphylococcal enterotoxin D gene 451 bp (Figure 1 (Fig. 1)).

The enterotoxigenic strains were tested for production of SEs type SEA, SEB, SEC and SED. All four types of SEs were produced by isolates from one or more meat product (Table 3 (Tab. 3)). Only minced beef produced all four types of SEs; beef burger, beef sausage and beef kofta produced three types while beef luncheon produced two types of SEs. 

## Discussion

Meat processed products from Saudi Arabia and Egypt were investigated for contamination with SEs in the present study. Staphylococcal food poisoning is a common disease whose incidence may be under-estimated for many reasons. As meat constitutes a vehicle for transmission of the disease, hence survey of SEs in meat contributes to its control and promotion of public health. 

The presence rate of *S. aureus* in examined minced meat, beef burger, beef sausage, beef kofta and beef luncheon, was 38%, 22%, 30%, 32% and 12%, respectively. As well the presence rate of enterotoxigenic *S. aureus* in examined minced meat, beef burger, beef sausage, beef kofta and beef luncheon was 16%, 8%, 8%, 6% and 2%, respectively. Some factors that may attribute to bacterial contamination of different meat types could be due to the way each type is processed and sort of meat, such as intestinal parts, included. Also, the addition of accessory ingredients to meat, such as vegetables and sauce could be another source of contamination. These results were in agreement with studies reported by [11] and [12]. Lower incidence was achieved by [13] and [14], but higher incidence was detected by [15] and [16]. Observed differences between results of the present study and above-mentioned studies may be explained by source of food, added ingredients, way of preparation and laboratory procedures. 

A total of 20 *S. aureus* strains from each of the meat product samples were tested for enterotoxin production. From minced meat, 8 strains produced toxin A, B, C and D while in strains isolated from beef burger, 4 strains only produce toxin A, B and C. In the strains isolated from beef sausage, 4 strains produced toxins A, B and D while in strains isolated from beef kofta, 3 strains only produced toxin A, B and C but in strains isolated from beef luncheon, 2 strains produce toxins, A and B only. These results were in agreement to some extent with the findings of [17] and [12]. The discrepancy may be due to the different methodology as the above-mentioned researchers used latex agglutination test for detection of *S. aureus* toxins.

In the present study, with a novel multiplex PCR to detect *S. aureus* super-antigens [10], enterotoxin genes could be detected at different bands of agarose gel. The obtained results are nearly similar to those reported by [10], [18], [19], [20], [21], who detected staphylococcal enterotoxins by multiplex PCR technique. The majority of reported SFP outbreaks are associated with the classical enterotoxins, SEA-SEE and staphylococcal enterotoxin A (SEA) being considered the most common cause of SFP [22], [23]. SEA is the most common enterotoxin recovered from food-poisoning outbreaks in the US (77.8% of all outbreaks) followed by SED (37.5%) and SEB (10%) [24].

Molecular bacteriology techniques should be used further, in the study area, to investigate all types of SEs to evaluate the effective hazards these toxins present to food safety. Food safety is one of the WHO’s 13 strategic objectives for 2008–2013. It is a very important approach in attempts to control foodborne diseases by health authorities in the world [6]. 

The enterotoxins are responsible for the symptoms of staphylococcal food poisoning and may have a role in the pathogenicity of some staphylococcal disease. The symptoms include nausea, vomiting and less frequently diarrhea, headache, dizziness and weakness [25]. 

In general, staphylococcal food poisoning symptoms are characterized by nausea, vomiting, diarrhea, general malaise, and weakness, beginning from 1 to 6 hours (usually 2 to 4 hours) after ingestion of food. Although the illness is rarely fatal complications may include dehydration and shock. Recovery usually occurs within 24 hours, however, occasionally may take up to several days [26].

Meat products may be responsible in many *S. aureus* poisoning outbreaks, while the contamination of such meat products may occur during handling and preparation or due to post processing contamination or they may be kept unrefrigerated for several hours, during such time *S. aureus* multiply and elaborate enterotoxin. Handling of ready-to-eat meat products leads to the addition of high amount of microorganisms, especially, potential food poisoning organisms as coagulase positive *S. aureus*. This contamination level is usually not affected by other factors, as mentioned by [27] and [28]. In general heat treatment of the food may eliminate contaminating bacteria, but the enterotoxins are very heat resistant. Thus, although the bacteria are killed, the toxins will remain and can cause food poisoning [28]. 

Because *S. aureus* does not normally compete well with indigenous microorganisms in raw foods, contamination is mainly associated with improper handling of cooked or processed foods, followed by storage under conditions which favour growth and multiplication of *S. aureus* and production of the enterotoxin(s). 

It could be concluded that *S. aureus* producing enterotoxins are present in beef products samples in the study area. Multiplex PCR could be used to detect classical types of SEs in different prepared foods.

## Notes

### Competing interests

The authors declare that they have no competing interests.

## Figures and Tables

**Table 1:**
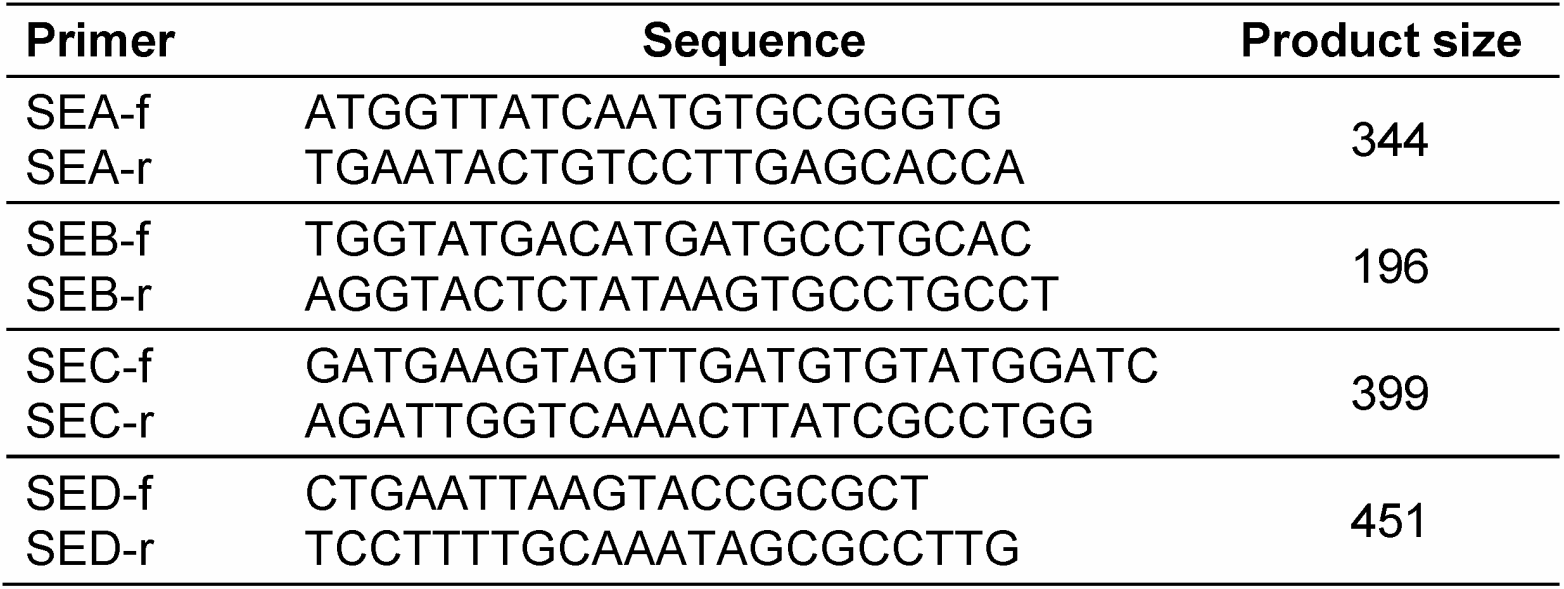
*S. aureus* gene-specific oligonucleotide primers sequence [10]

**Table 2:**
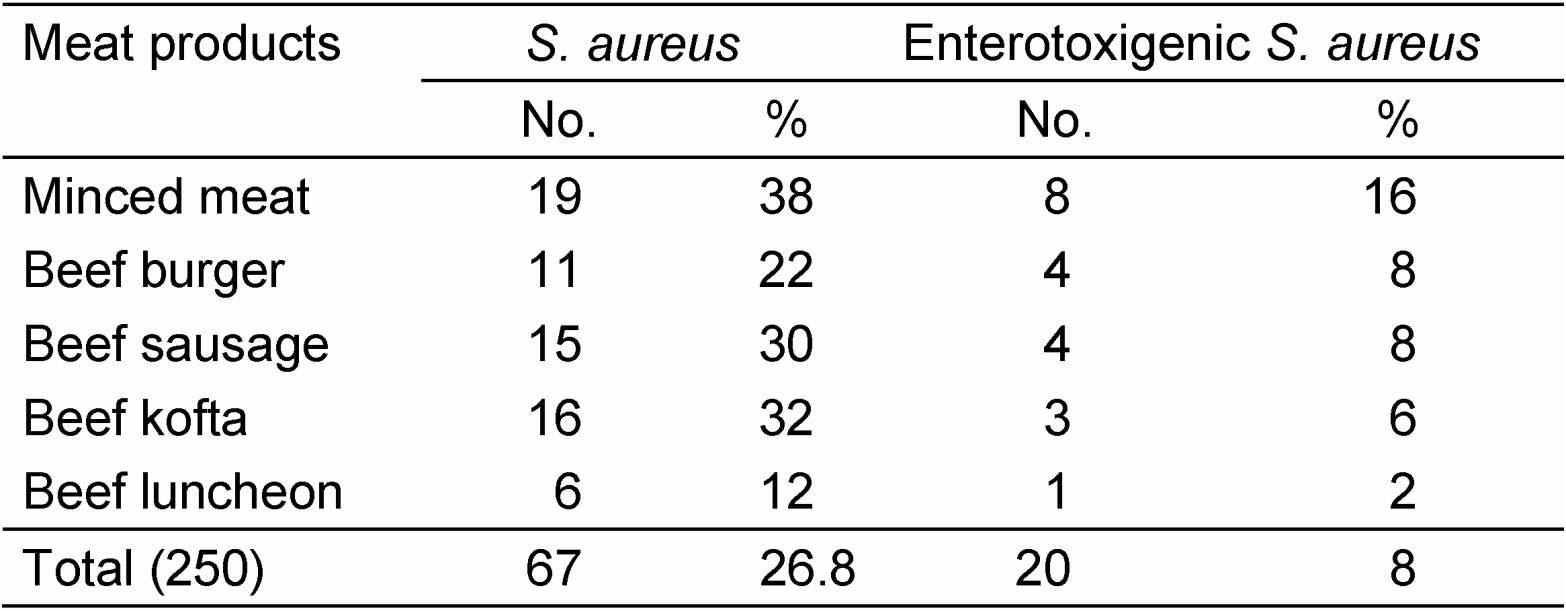
Prevalence of enterotoxigenic *S. aureus* in the examined meat products

**Table 3:**
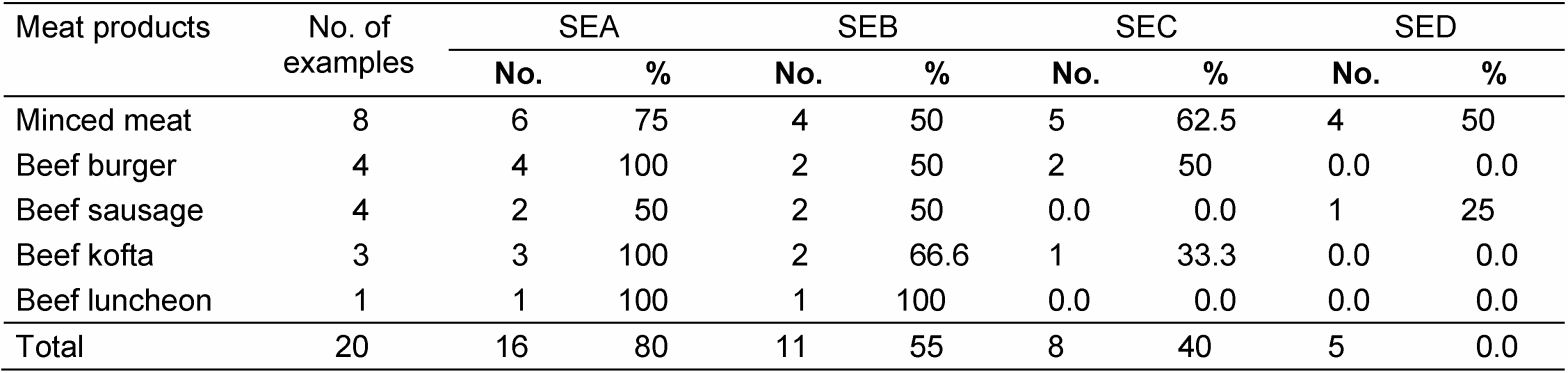
Type of enterotoxins produced by isolated strains of *S. aureus*
